# Enhancing particle bunch-length measurements based on Radio Frequency Deflector by the use of focusing elements

**DOI:** 10.1038/s41598-020-67997-1

**Published:** 2020-07-10

**Authors:** Pasquale Arpaia, Roberto Corsini, Antonio Gilardi, Andrea Mostacci, Luca Sabato, Kyrre N. Sjobak

**Affiliations:** 1 University of Naples Federico II, DIETI - IMPALab, Napoli, Italy; 2 CERN, BE, Geneva, Switzerland; 3 University of Rome La Sapienza, Rome, Italy; 4 University of Oslo, Oslo, Norway

**Keywords:** Electrical and electronic engineering, Particle physics, Techniques and instrumentation

## Abstract

A method to monitor the length of a particle bunch, based on the combination of a Radio Frequency Deflector (RFD) with magnetic focusing elements, is presented. With respect to state-of-the-art bunch length measurement, the additional focusing element allows to measure also the correlations between the longitudinal and transverse planes in terms of both position and divergence. Furthermore, the quadrupole-based focusing increases the input dynamic range of the measurement system (i.e.  allows for a larger range of beam Twiss parameters at the entrance of the RFD). Thus, measurement resolution and precision are enhanced, by simultaneously preserving the accuracy. In this paper, the method is first introduced analytically, and then validated in simulation, by the reference tool ELEctron Generation ANd Tracking, ELEGANT. Finally, a preliminary experimental validation at CLEAR (CERN Linear Electron Accelerator for Research) is reported.

## Introduction

**Figure 1:**
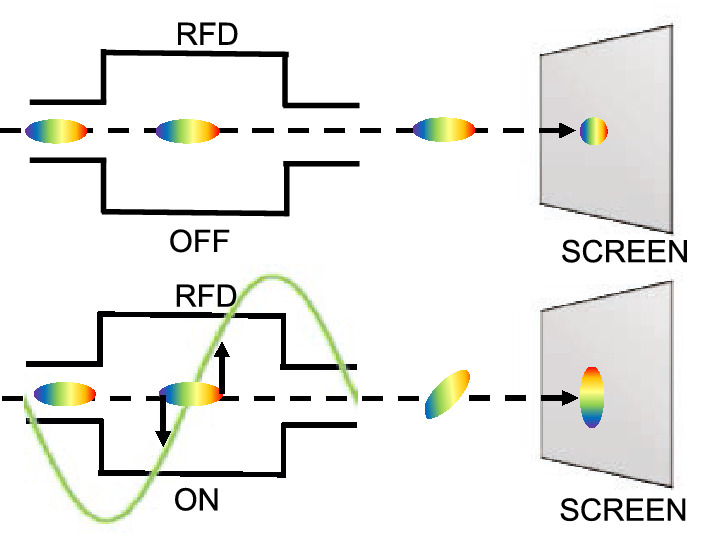
Operation with RFD off (top) and on (bottom): effect on the beam.

In monitoring LINear ACcelerators (LINACs), one of the main parameters to be precisely measured is the bunch length. One of the most common method exploits a transverse deflecting structure^1,2^, namely a Radio Frequency Deflector (RFD)^3–5^. The method operation is highlighted in Fig. 1. Initially, the RFD is off (Fig. 1, top). For the measurement (Fig. 1, bottom), a time-dependent transverse kick is given to the electron bunch^6^.

In this way, the longitudinal and transverse bunch dimensions of the beam on the screen are correlated. The bunch length can then be obtained from the measurement of the spot size in the direction of the deflection, after a suitable calibration of the displacement dependence on the deflecting voltage RF phase^7^. The method allows to measure ultra-short electron beam bunches down to few fs^1,3,8,9^. The combination of an RFD and a dispersive element (i.e.  a dipole) can be exploited to measure the longitudinal phase space distribution of the beam^7,10^.

RFDs are widely used in LINACs around the world, owing to their very high resolutions. For example, RFDs are used at CERN Linear Electron Accelerator for Research (CLEAR)^11^, Stanford Linear Accelerator Center (SLAC)^12,13^, Deutsches Elektronen–Synchrotron (DESY)^14^, Massachusetts Institute of Technology (MIT) Plasma Science and Fusion Center (PSFC)^15^, Sources for Plasma Accelerators and Radiation Compton with Lasers And Beams (SPARC LAB)^16,17^, the ultraviolet and soft X-ray FEL facility Free-electron LASer in Hamburg (FLASH)^18^, the Accelerator Test Facility (ATF) in Brookhaven National Lab (BNL)^19^, and so on.

Other well-known techniques to perform bunch length measurements are: (i) the streak camera: a high time resolution camera (based on the use of a photo-multiplier and a rapidly changing electric field) collects the light from a specific screen (e.g.  an Optical Transition Radiation screen) which produces a light pulse of the same length as the electron bunch^20^; (ii) Electro-Optical Sampling (EOS), which uses an external laser that passes through a non linear crystal parallel to the beam, to measure the polarization modulation on the laser pulse due to the electric field associated with the bunch^21,22^; (iii) spectral analysis of the bunch frequency content; the bunch length is assessed by analyzing the frequency spectrum of coherent emission from the bunch^23–27^, and (iv) the measurement of the beam energy spread when using an accelerating cavity not on the RF crest^28–30^.

In various accelerators, focusing elements are installed between the RFD and the screen (e.g.  CLEAR^11^, ATF^31^, and DESY^32^) for various reasons. Usually, the space between the RFD and the screen is just needed to deflect enough the bunch (Fig. 1). In other cases, dimensional problems arise in the physical installation as a whole, e.g. to reduce the space between the klystron and the deflecting cavity, by consequently shortening the necessary waveguides. Furthermore, quadrupoles could be used to focus the beam on the screen. However, the impact on the RFD measurement quality of the position of the focusing element was not investigated until now.

In this paper, a theoretical derivation of the bunch length measurement, when focusing elements are present between the RFD and the screen, is presented. It will be proven that the focusing element does not introduce additional effects that can invalidate the measurement. Furthermore, the metrological performance of the method with the focusing element is analyzed.

In particular, in section “Methods”, the general theory is derived analytically. In section “Validation using tracking simulations”, the mathematical derivation is numerically validated through a reference simulation tool, the ELEGANT code^33^. In section “Experimental verification”, a preliminary experimental validation on CLEAR at CERN is reported. Finally, in “Discussion”, the promising advantages of the non-conventional layout are discussed.

## Methods

**Figure 2:**
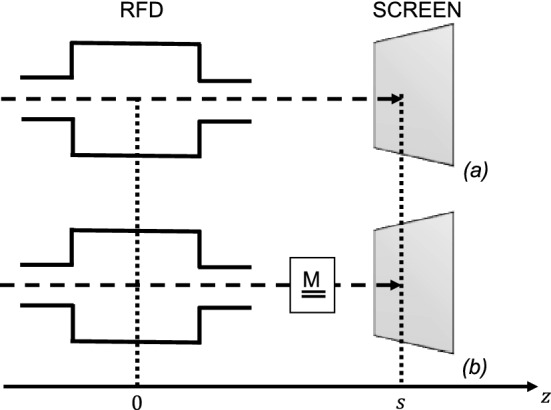
Layouts of an RFD-based measurement: (**a**) conventional, without elements between the RFD and the screen; and (**b**) non-conventional, with a generic linear element described by the matrix $\underline{{\underline{M}}}$.

In the following, the term “conventional layout” of an RFD-based measurement points to the configuration without a focusing element (Fig. 2a), while “non-conventional” to its presence (Fig. 2b). In the conventional layout, some focusing elements (e.g.  quadrupoles) are usually placed before the RFD, and can be used to minimize the beam spot on the screen and to improve the measurement resolution^4,34^. In the non-conventional layout, additional focusing elements are also placed between the RFD and the screen.

In this section, the analytical equations for the centroid and the vertical spot size with the RFD turned on and off are derived for the non-conventional layout. The analytical treatment is first carried out for a generic linear element (i.e.  described through a matrix $\underline{{\underline{M}}}$) between the RFD and the screen, and then applied to the quadrupole case. For the sake of the simplicity, the analysis is carried out for the vertical plane. But, an analogous analytical treatment is also valid for the horizontal plane. Finally, the measurement method is formalized.

### RFD OFF

As usual in literature, the deflector is approximated as a thin element and the transverse kick is applied at the center of the RFD^34^. In the case of RFD turned off, the equations describing the system behavior in terms of positions (*y*) and divergences ($y'$) are:

1 $$\begin{aligned} \left( \begin{array}{cc} y_{s,OFF}\\ y'_{s,OFF} \end{array}\right) = \left( \begin{array}{cc} M_{11} &{} M_{12}\\ M_{21} &{} M_{22} \end{array}\right) \left( \begin{array}{cc} y_0\\ y^{\prime}_0 \end{array}\right) , \end{aligned}$$

the subscripts *s* and 0 are for the variables at the screen and the RFD center, respectively (as shown in Fig. 2). The terms $M_{ij}$ are the elements of the transport matrix $\underline{{\underline{M}}}$ of the components between the RFD and the screen. The vertical spot size at the screen, with RFD off, can be calculated using Eq. 1:

2 $$\begin{aligned} \sigma ^2_{y_{s,OFF}} = \langle y^2_{s,OFF}\rangle -\langle y_{s,OFF}\rangle ^2 = M^2_{11}\sigma ^2_{y_0}+M^2_{12}\sigma ^2_{y^{\prime}_0}+2M_{11}M_{12}\sigma _{y_0y^{\prime}_0}, \end{aligned}$$

where $\sigma _{y_0}$ is the initial rms dimension of the bunch, $\sigma _{y^{\prime}_0}$ is the initial rms divergence, and $\sigma _{y_0{y^{\prime}_0}}~=~\langle (y_0 -\langle y_0\rangle )( y^{\prime}_0-\langle y^{\prime}_0\rangle )\rangle$ is the correlation between position and divergence, all in the vertical plane.

### RFD ON

In the case of RFD on, the system equation becomes:

3 $$\begin{aligned} \left( \begin{array}{cc} y_{s,ON}\\ y'_{s,ON}\end{array}\right) = \left( \begin{array}{cc} M_{11} &{} M_{12}\\ M_{21} &{} M_{22} \end{array}\right) \left( \begin{array}{cc} y_0\\ y^{\prime}_0+\Delta y^{\prime}_0 \end{array}\right) , \end{aligned}$$

where $\Delta y^{\prime}_0$ represents the transverse kick given by the deflector in the short deflector and bunch approximation ( $\sin (kz_0+\phi )= \sin (kz_0)\cos (\phi )+\cos (kz_0)\sin (\phi )\approx kz_0\cos (\phi ))+\sin (\phi )$)^34^:

4 $$\begin{aligned} \Delta y^{\prime}_0(z_0,\phi ) = \frac{V_t}{E_0}\sin (kz_0+\phi ) \approx \frac{V_t}{E_0}kz_0\cos (\phi )+\frac{V_t}{E_0}\sin (\phi ), \end{aligned}$$

where $E_0$ is the energy of the reference particle, $z_0$ the displacement between the position of the particle and the center of the beam in the longitudinal plane in the laboratory reference frame, *k* the wavenumber ($k = {2 \pi f_{RF}}/{c}$) of the deflecting voltage, and $V_t$, $\phi$, and $f_{RF}$ are the amplitude, the RF phase, and the frequency, respectively, of the transverse deflecting voltage.

#### Centroid position

The beam in terms of distribution is assessed by introducing the centroid, i.e.  the average vertical position of the electrons over the whole bunch. The variation of the centroid position with the RF phase $C_{y_{s,ON}}(\phi )$ can be written as:

5 $$\begin{aligned} C_{y_{s,ON}}(\phi ) = \langle y_{s,ON}\rangle = \langle M_{11}y_0\rangle +\langle M_{12}y^{\prime}_0\rangle +\langle M_{12}\Delta y_0'\rangle . \end{aligned}$$

By writing Eq. 1, it is already assumed that the elements $M_{ij}$ are identical for all particles. In the specific case of single quadrupole, this assumption means that the energy spread is neglected. The terms $M_{ij}$ can be extracted from the average:

6 $$\begin{aligned} C_{y_{s,ON}}(\phi ) = M_{11}\langle y_0\rangle +M_{12}\langle y^{\prime}_0\rangle +M_{12} \frac{V_t}{E_0}k\cos (\phi )\langle z_0\rangle +M_{12}\frac{V_t}{E_0}\sin (\phi ). \end{aligned}$$

Let’s assume that $\langle y_0 \rangle = 0$, $\langle y^{\prime}_0 \rangle = 0$, and $\langle z_0 \rangle = 0$. Thus, the vertical centroid at the screen becomes:

7 $$\begin{aligned} C_{y_{s,ON}}(\phi ) = M_{12}\frac{V_t}{E_0}\sin (\phi ). \end{aligned}$$

#### Calibration factor

A calibration factor $K_{CAL}$ can be defined^1^:

8 $$\begin{aligned} K_{CAL}(\phi ) = 2\pi f_{RF}\frac{\mathrm {d} C_{y_{s,ON}}(\phi )}{\mathrm {d}\phi }. \end{aligned}$$

$K_{CAL}$ allows to assess the bunch length from the vertical beam size and from the frequency of the deflector $f_{RF}$. The calibration is done by varying the RF phase of the RFD and observing the effect on the centroid on the screen^7^. From Eqs. 7 and 8, the calibration factor can be determined as:

9 $$\begin{aligned} K_{CAL}(\phi ) = 2\pi f_{RF}M_{12}\frac{V_t}{E_0}\cos (\phi ) \end{aligned}$$

The Eq. 9 is a general form of the conventional expression of the calibration factor in^35^.

#### Vertical spot size

Switching on the RFD, the vertical spot on the screen is calculated using Eqs. 3 and 4:

10 $$\begin{aligned} \begin{aligned} \sigma ^2_{y_{s,ON}}(\phi )&= M^2_{11}\sigma ^2_{y_0}+M^2_{12}\sigma ^2_{y^{\prime}_0}+2M_{11}M_{12}\sigma _{y_0{y^{\prime}_0}} +M^2_{12}\left( \frac{V_t}{E_0}k\cos (\phi )\right) ^2c^2\sigma ^2_{t_0}+ 2M_{11}M_{12}\frac{V_t}{E_0}kc\cos (\phi )\sigma _{y_{0}t_{0}} \\&\quad +2M^2_{12}\frac{V_t}{E_0}kc\cos (\phi )\sigma _{y^{\prime}_0t_0}. \end{aligned} \end{aligned}$$

All the terms of $\sigma ^2_{y_{s,OFF}}$ (Eq. 2) are present in Eq. 10, plus additional terms related to: (i) the bunch length $\sigma _{t_0}$ (i.e.  the value to be measured), and the correlation between longitudinal position and the vertical plane ($\sigma _{y_{0}t_{0}}$ and $\sigma _{y^{\prime}_0t_0}$). Contracting the expression using $\sigma ^2_{y_{s,OFF}}$ and $K_{CAL}(\phi )$ (Eq. 9), $\sigma ^2_{y_{s,ON}}$ can be written as:

11 $$\begin{aligned} \sigma ^2_{y_{s,ON}}(\phi ) = \sigma ^2_{y_{s,OFF}}+K^2_{CAL}(\phi )\sigma ^2_{t_0}+2M_{11}K_{CAL}(\phi )\sigma _{y_{0}t_{0}}+ 2M_{12}K_{CAL}(\phi )\sigma _{y^{\prime}_0t_0}. \end{aligned}$$

Also in this case, Eq. 11 is a general expression of the formula found in^35^. In the case of conventional layout ($M_{11}~=~1$ and $M_{12}~=~L$), Eqs. 9 and 11, provide the $K_{CAL}$ and the $\sigma _{y_{s,ON}}^2$ , respectively^35,36^. The terms due to the correlations can be canceled by averaging two measurements in phase opposition:

12 $$\begin{aligned} \overline{\sigma _{y_{s,ON}}}^2(\phi ) = \frac{\sigma ^2_{y_{s,ON}}(\phi )+ \sigma ^2_{y_{s,ON}}(\phi +\pi )}{2}= \sigma ^2_{y_{s,OFF}}+K^2_{CAL}(\phi )\sigma ^2_{t_0}. \end{aligned}$$

The term $\overline{\sigma _{y_{s,ON}}}^2$ is equal to $\sigma ^2_{y_{s,ON}}$ in absence of correlations between the vertical and longitudinal planes.

From Eqs. 9, 11, and 12, some preliminary points can be made: (i) a calibration factor can be defined with the same meaning of the conventional layout (i.e.  including the variation of the centroid on the screen); (ii) the non-conventional layout does not introduce any deterministic error source in the measurement, (iii) the possibility of removing the correlation effects (i.e.  $\sigma _{y^{\prime}_0t_0}$,and $\sigma _{y_{0}t_{0}}$) is preserved, by carrying out two different measurements of $\sigma _{y_{s,ON}}$ in phase opposition (at $\phi$ and $\phi + \pi$), and then evaluating the average between their squared values^35,36^, and (iv) thanks to the dependence of $\sigma _{y_{s,ON}}$ and $\sigma _{y_{s,OFF}}$ on the focal length, these quantities can be optimized over a wider range of beam parameters.

### Single quadrupole case

**Figure 3:**
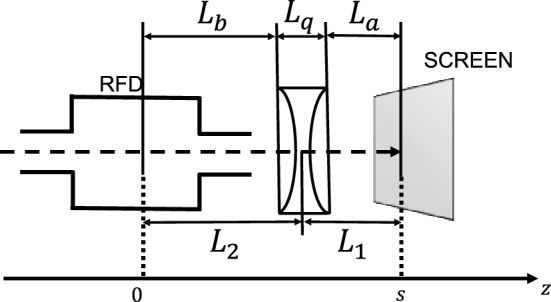
An example of a non-conventional layout, with a vertical focusing quadrupole between the RFD and the screen.

In the case of a single thick focusing quadrupole, the transport matrix $\underline{{\underline{M}}}$, between the RFD and the screen, is obtained from the multiplication of the following matrices:

13 $$\begin{aligned} \underline{{\underline{M}}} = \left( \begin{array}{cc} 1 &{} L_a\\ 0 &{} 1 \end{array}\right) \left( \begin{array}{cc} \cos (\sqrt{k}L_q) \frac{\sin (\sqrt{k}L_q)}{\sqrt{k}}\\ \sqrt{k}\sin (\sqrt{k}L_q) \cos (\sqrt{k}L_q) \end{array}\right) \left( \begin{array}{cc} 1 &{} L_b\\ 0 &{} 1\end{array}\right) , \end{aligned}$$

where *k* is the focusing strength of a quadrupole, $L_q$ the effective magnetic length of the quadrupole, $L_a$ the drift space from the end of the quadrupole to the screen, and $L_b$ the drift from the middle of the RFD to the entrance of the quadrupole (see Fig. 3).

In order to do some considerations, it is convenient to use the well-known thin lens approximation. The matrix $\underline{{\underline{M}}}$ becomes:

14 $$\begin{aligned} \underline{{\underline{M}}} = \left( \begin{array}{cc} 1 &{} L_1\\ 0 &{} 1 \end{array}\right) \left( \begin{array}{cc} 1 &{} 0\\ -\frac{1}{f} &{} 1 \end{array}\right) \left( \begin{array}{cc} 1 &{} L_2\\ 0 &{} 1 \end{array}\right) , \end{aligned}$$

where *f* is the quadrupole focal length, $L_1$ is the distance between the quadrupole center and the screen (i.e.  $L_1 = L_a+\frac{L_q}{2}$), and $L_2$ is the distance between the RFD center and the quadrupole center (i.e.  $L_2~=~L_b+\frac{L_q}{2}$). The relation between the focusing strength and the focal length is:

15 $$\begin{aligned} f = \frac{1}{kL_q}. \end{aligned}$$

For a focusing magnet in the plane of interest (i.e.  vertical) *f* is positive, while in the same plane for a defocusing magnet *f* is negative.

With this approximation, the terms $M_{11}$ and $M_{12}$ are:

16 $$\begin{aligned} M_{11}= & {} 1-\frac{L_1}{f} \end{aligned}$$

17 $$\begin{aligned} M_{12}= & {} (L_1+L_2)-\frac{L_1L_2}{f} \end{aligned}$$

In the non-conventional layout, the term $M_{12}$ is present in $K_{CAL}(\phi )$ (Eq. 9), which depends on the focal length of the quadrupole (from Eq. 17). Therefore, $\sigma _{y_{s,OFF}}$ (depends as well on the focal length Eq. 2) cannot be decorrelated from the deflecting power as in the conventional layout.

Another result that comes from Eq. 17 is the possibility to make $M_{12}$ zero, and consecutively also $K_{CAL}(\phi )$ (Eq. 9). This happens when the focal length is equal to

18 $$\begin{aligned} f^* \equiv \frac{L_1L_2}{L_1+L_2}\;. \end{aligned}$$

From a physical point of view, operating with such a value of focal length means that the RFD is increasing the bunch spot size on the screen by exactly the same amount as the quadrupole is squeezing it. There is a phase advance of $\pi$ between the $z=0$ and $z=s$. Therefore, for this value of focal length, the measurement cannot be carried out.

All the derived equations and considerations can be extended for a general configuration such as a doublet or triplet.

### Measurement method

From Eq. 12, considering $\phi = {0}\hbox {rad}$, the formula for the bunch length can be written in the same form as in literature^34^:

19 $$\begin{aligned} \sigma _{t_{0,m}} = \sqrt{\frac{\overline{\sigma _{y_{s,ON}}}^2-\sigma ^2_{y_{s,OFF}}}{K_{CAL}^2}}. \end{aligned}$$

In Eq. 19, the measured values are $\overline{\sigma _{y_{s,ON}}}$ and $\sigma _{y_{s,OFF}}$, while the measurand is $\sigma _{t_0}$. If the values of $\sigma _{y_{s,OFF}}$ and $\sigma _{y_{s,ON}}$ are too close, the information on the measurand could be lost in the uncertainty or in the resolution of the measurement.

## Validation using tracking simulations

The aim of this section is to validate numerically the model proposed in section “Methods”, by exploiting the parameters of the machine CERN Linear Electron Accelerator for Research (CLEAR)^11^. Several simulations were carried out by scanning the focal length of the quadrupole. All the equations were tested by taking into account the correlations between longitudinal and vertical planes. Then, the simulation results were compared with the ones of the analytical equations in the previous section.

### Simulation setup

Tracking codes (e.g.  ELEGANT, ASTRA, MAD-X, and so on) are very useful and reliable tools in order to simulate the behavior of particle bunches in accelerators^37^. These codes have been validated against a large number of different practical cases, and are currently used as reference standards in designing and commissioning modern accelerators.

Both the cases of conventional and non-conventional layout were simulated by referring to the configuration of the accelerator CLEAR. In particular, the used parameters and their corresponding values are reported in Table 1, where: $\alpha _y$ and $\beta _y$ are the Twiss parameters at the entrance of the RFD; $\epsilon _{g,y}$ is the beam geometrical emittance; $\sigma _{t_0}$ is the bunch length; $L_a$, $L_b$, and $L_{q}$ correspond to the lengths shown in Fig. 3; $L_{RFD}$ is the RFD length; $V_t$ and $f_{RF}$ are the amplitude and frequency of the deflecting voltage, respectively; $f_{min}$ is the minimum focal length and depends on the beam stiffness and the type of quadrupole (the quadrupole parameters correspond to the QL3 magnets used in CLEAR^38^); and $f^*$ is the focal length to zero the calibration factor (reported in Eq. 18). The total distance between the RFD and the screen was kept constant in the simulations.

**Table 1:** CLEAR-like parameters used in the ELEGANT simulations, where: $\alpha _y$ and $\beta _y$ are the Twiss parameters at the entrance of the RFD; $\epsilon _{g,y}$ is the beam geometrical emittance; $\sigma _{t_0}$ is the bunch length; $L_a$, $L_b$, and $L_{q}$ correspond to the lengths shown in Fig. 3; $L_{RFD}$ is the RFD length; $V_t$ and $f_{RF}$ are the amplitude and frequency of the deflecting voltage, respectively; $f_{min}$ is the minimum focal length achievable by the quadrupole; and $f^*$ is the focal length to zero the calibration factor (reported in Eq. 18).

Parameters	Value
$\alpha _y$	3
$\beta _y$ (m)	10
$\epsilon _{g,y}$ (nm)	10
Energy (MeV)	220
Bunch charge (pC)	50
$\sigma _{t_0}$ (ps)	1
Energy spread	5$\%$
$L_a$ (m)	0.887
$L_b$ (m)	0.887
$L_{q}$ (m)	0.226
$L_{RFD}$ (m)	0.116
$V_{t}$ (MV)	10
$f_{RF}$ (GHz)	2.998
$|f_{min}|$ (m)	0.2899
$f^*$ (m)	0.443
Macro particles	500,000

### Simulation results

**Figure 4:**
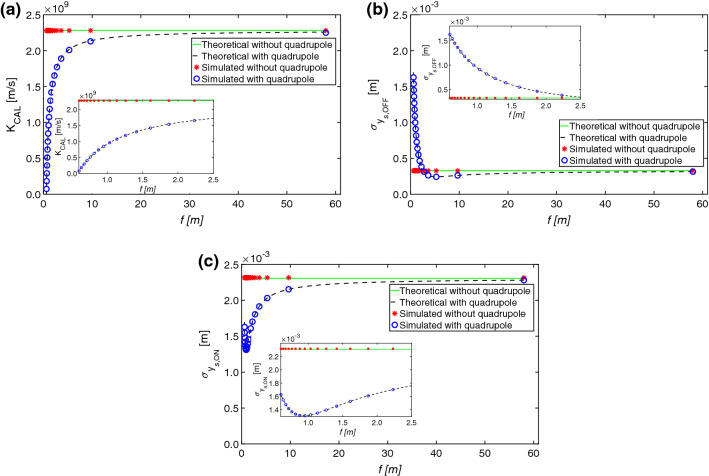
Comparison between theoretical values (solid line without quadrupole, and dashed line with quadrupole) and simulation results (stars without quadrupole, and dots with quadrupole) for: (**a**) $K_{CAL}$ (Eq. 8), (**b**) $\sigma _{y_{s,OFF}}$ (Eq. 2), and (**c**) $\sigma _{y_{s,ON}}$ (Eq. 11), versus the focal length (zoom at low *f* on the side).

The comparisons between theoretical values (solid and dashed lines) and the simulation results (stars and dots line) for $K_{CAL}$, $\sigma _{y_{s,OFF}}$, and $\sigma _{y_{s,ON}}$ are shown in Fig. 4a–c respectively.

The dashed lines and the dots are used for the case of the conventional layout, while the solid lines and the stars for the non-conventional layout.

For the conventional layout, the values of the $K_{CAL}$, $\sigma _{OFF}$, and $\sigma _{ON}$ do not depend on the focal length, owing to the absence of the quadrupole. Conversely, for the non-conventional layout, the values change with the focal length of the quadrupole. For the sake of the simplicity, only the case of the focusing quadrupole is reported, but also the defocusing case was tested analogously. The minimum focal length used in the simulations is $f^*$, derived from Eq. 18. Its numerical value is shown in the row before the last of Table 1. The Twiss parameter used (see Table 1) position the waist after the screen. The curve of the $K_{CAL}$ (for $\phi = 0$) of the non-conventional layout tends to the curve of the conventional layout while at increasing the focal length, (i.e.  switching off the quadrupole, see Fig. 4a). This can be explained from Eq. 9, which contains $M_{12}$ from Eq. 17. Switching off the quadrupole is equivalent to let *f* approach infinity. The maximum calibration factor is obtained in the absence of the focusing quadrupole.

In Fig. 4b, all the points of the solid line (i.e.  $\sigma _{y_{s,OFF}}$ in the non-conventional layout) above the dashed line (i.e.  $\sigma _{y_{s,OFF}}$ in the conventional layout) correspond to over-focusing, where the quadrupole is making the beam on the screen larger than in the case without quadrupole. On the other hand, there are values of focal length that make $\sigma _{y_{s,OFF}}$ smaller than the value obtained with the conventional layout. The minimum of $\sigma _{y_{s,OFF}}$ corresponds to the focal length that imposes the waist at the screen position.

In the considered range, all the values of $\sigma _{y_{s,ON}}$ of the non-conventional layout are smaller than the values of the conventional layout for all the focal lengths, as seen in Fig. 4c.

A satisfying agreement between the theory and the simulation is achieved: for $K_{CAL}$, $\sigma _{OFF}$, and $\sigma _{ON}$, the maximum difference is less than $0.6\%$. From Eq. 19 it is possible to see that to have a good measurements a relatively large difference between $\sigma _{y_{s,ON}}$ and $\sigma _{y_{s,OFF}}$ would be needed. Thanks to the flexibility added by additional focusing elements it is easier to achieve these conditions. The relative error, between the theoretical $\sigma _{t_0}$ and the simulated $\sigma _{t_{0,m}}$ values, using Eq. 19, is defined as:

20 $$\begin{aligned} \epsilon _{\%} = 100 \cdot \frac{|\sigma _{t_0}-\sigma _{t_{0,m}}|}{\sigma _{t_0}}. \end{aligned}$$

**Figure 5:**
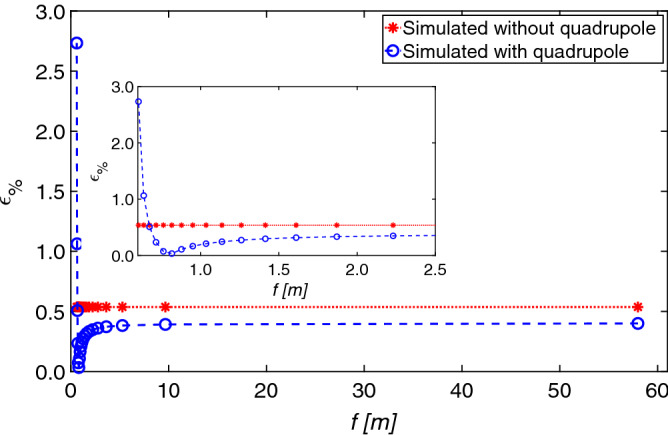
Relative error of the bunch length versus the focal length (Eq. 20): theoretical values (dotted line without quadrupole, and dashed line with quadrupole) and simulation results (stars without quadrupole, and dots with quadrupole).

The error is smaller than $0.5\%$ (Fig. 5).

A simulation was performed using $f = f^*$ (as defined in Eq. 18), by imposing $\sigma _{y_{s,ON}} = \sigma _{y_{s,OFF}}$. In this case, the bunch length cannot measured, because no information on the measurand can be obtained from the size of the bunch.

In the simulations, for *f* approaching $f^*$, the error is still lower than $3\%$, but this is an artifact because the resolution of the screen is not taken into account.

### Correlations between longitudinal and transverse position

**Figure 6:**
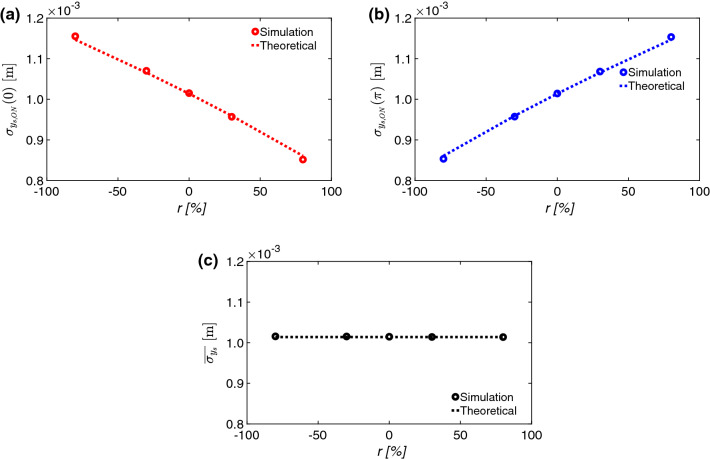
(**a**) $\sigma _{y_{s,ON}}(0)$, (**b**) $\sigma _{y_{s,ON}}(\pi )$, and (**c**) $\sigma _{y_{s}}$ versus *r* (theoretical results from Eq. 11 for (**a**) and (**b**) and from Eq. 12 for (**c**) in dashed lines, and simulations results in dots).

In this section, the effects of the correlations between the particle longitudinal positions and the position/divergence in the vertical plane are analyzed. In particular, the correlation terms of Eq. 11 (proportional to $\sigma _{y_0 t_0}$ and $\sigma _{{y^{\prime}_0} t_0}$) are compared with the simulation results. Furthermore, the cancellation of the correlation terms is validated with simulations (see Eq. 12) in Fig. 6.

The correlation terms between the planes are defined as:

21 $$\begin{aligned}&\sigma _{y_{0}t_{0}}= r_{y_0t_0}\sigma _{y_0}\sigma _{t_0}\\& \sigma _{y^{\prime}_0t_0}=r_{y^{\prime}_0t_0}\sigma _{y^{\prime}_0}\sigma _{t_0}, \end{aligned}$$

where $r_{y_{0}t_{0}}$ and $r_{y^{\prime}_0t_0}$ are the correlation factors between the particle longitudinal positions and the vertical positions, and between the particle longitudinal positions and the vertical divergences, respectively.

Simulations at two different RF phases (${0}\hbox {rad}$ and ${\pi }\hbox {rad}$), and varying the correlation coefficients $r_{y_{0}t_{0}}$ and $r_{y^{\prime}_0t_0}$, are shown in Fig. 6a and b, respectively. In the figure, the correlation coefficients are scanned, with the RFD phase equal to ${0}\hbox {rad}$ and ${\pi }\hbox {rad}$. In the scan, the correlation coefficients are equal, and the notation is simplified correspondingly: $r_{yt} = r_{y't} = r$.

Five correlation coefficients were chosen ($-80\%$, $-30\%$, $0\%$, $30\%$, $80\%$). In Fig. 6a and b, $\sigma _{y_{s,ON}}(0)$ and $\sigma _{y_{s,ON}}(\pi )$ are plotted respectively (theoretical results from Eq. 11 with dashed line and simulation results with dots). The value of $\sigma _{y_{s,ON}}(0)$ and $\sigma _{y_{s,ON}}(\pi )$ are only equal when the correlation coefficients are zero. In Fig. 6c, the average of the squared value between $\sigma _{y_{s,ON}}(\pi )$ and $\sigma _{y_{s,ON}}(0)$ is shown (Eq. 12). A satisfying agreement between theoretical values and simulation results (error less than 1%) was experienced.

## Experimental verification

A preliminary experimental validation at the CERN facility CLEAR was carried out. The preliminary experimental campaign consist of twofold main test sessions, with the quadrupole off and on, corresponding to both the conventional and non-conventional layouts: (i) verify that, in the same beam conditions, compatible values of bunch length are measured, and (ii) compare theoretical, simulation, and measurement results, for the vertical centroid position versus RF phase.

### CLEAR case study

The proposed method was implemented in CLEAR, an electron linear accelerator located at CERN^39^. CLEAR mainly aims to general accelerator research and development, as well as to component studies for existing and future accelerators.

CLEAR is based on a broad internal and external user community^39^. CLEAR was used as case study for the measurement method proposed here, because a quadrupole triplet is installed between the RFD and the screen.

**Figure 7:**
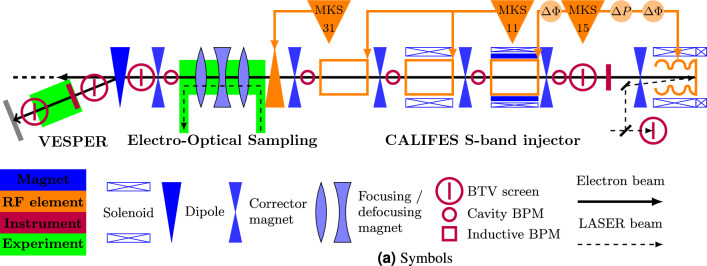
CLEAR injector layout with the location of the experimental stations^40^.

**Figure 8:**
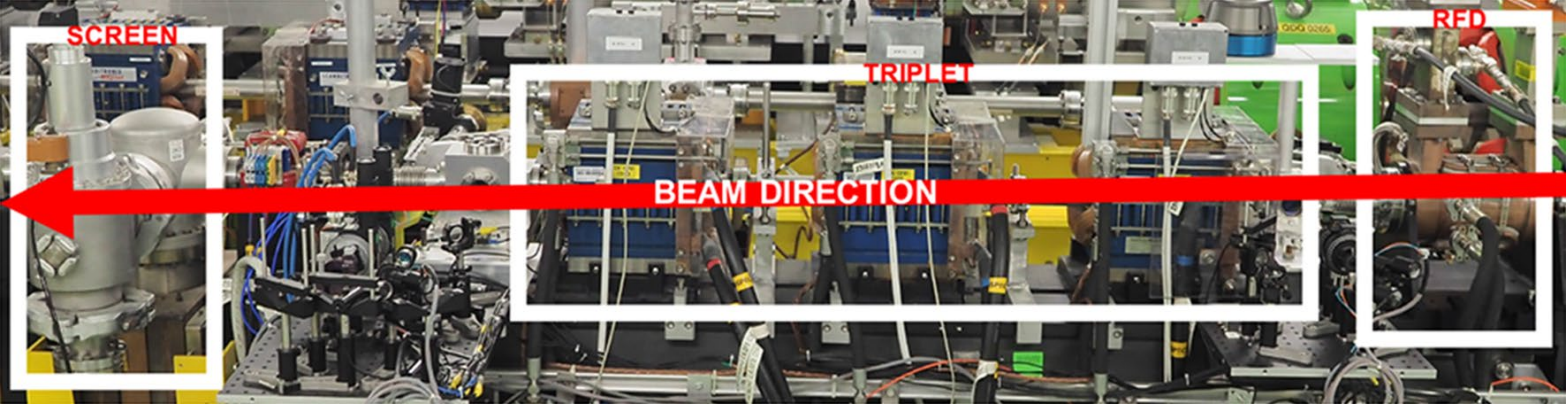
CLEAR beam-line: RFD (on the right), triplet (in the middle), and screen (on the left).

The relevant part of the layout of the CLEAR machine is shown in Fig. 7. A more detailed view of the location, where the RFD is installed, is shown in Fig. 8. The main parameters of the CLEAR machine are summarized in Table 2.

**Table 2:** CLEAR machine parameters, where: $\epsilon _{g,y}$ is the beam geometrical emittance; $\beta _y$ and $\alpha _y$ are the Twiss parameters at the entrance of the RFD; $L_a$, $L_b$, and $L_{q}$ correspond to the lengths shown in Fig. 3.

Parameters	Range
Energy (MeV)	60–220
Bunch charge (pC)	5–2000
Bunch length (ps)	0.2–5
$\epsilon _{g_{xy}}$ (nm)	1–20
$\beta _{x,y}$ (m)	1–100
$\alpha _{x,y}$	$-$7 to 7
Repetition rate (Hz)	1–10
Number of bunches in train	1–150
Bunch spacing (GHz)	1.5
$L_a$ (m)	0.800
$L_b$ (m)	1.20
$L_{q}$ (m)	0.226

The particle beam is described by the Twiss parameters measured at the entrance of the first quadrupole in the beam line.

### Measurement validation

In this section, the results of the experimental validation are shown for the conventional and non-conventional layout. The explored RF phase range is ${0.174}\hbox {rad}$. The current in the quadrupole is ${58} \hbox {A}$, corresponding to a focal length of 1m, chosen intentionally far from the value of $f^*$ (from Eq. 18). In the measurements, the bunch energy is ${200} \hbox {MeV}$, the charge ${80} \hbox {pC}$, and the deflecting voltage ${3.9} \hbox {MV}$. When the quadrupole is off (conventional layout), $\sigma _{y_{s,OFF}} = {183}\upmu \hbox {m}$, while when on (non-conventional layout), $\sigma _{y_{s,OFF}} = {147}\upmu \hbox {m}$.

**Figure 9:**
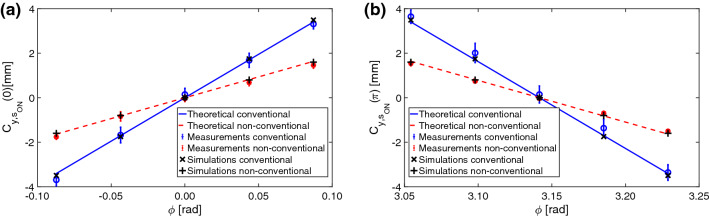
Vertical ($C_{y_{s,ON}}$) versus RFD phase in conventional and non-conventional layout around ${0} \hbox {rad}$ (**a**) and ${\pi } \hbox {rad}$ (**b**): measurements (circle and star for conventional and non-conventional layout, respectively), theoretical values (solid and dashed lines for conventional and non-conventional layout), and simulation points (cross and plus sign for conventional and non-conventional, layout respectively).

In Fig. 9a and b, the vertical centroid ($C_{y_{s,ON}}$) versus the RFD phase is shown around ${0} \hbox {rad}$ and ${\pi } \hbox {rad}$, respectively, for both the conventional and non-conventional layout. The measurements are pointed out with points (circle and star for conventional and non-conventional layout, respectively), the theoretical values with lines (solid and dashed for conventional and non-conventional layout, respectively), and the simulations with points (cross and plus sign for conventional and non-conventional layout, respectively). The 1-sigma repeatability bar for each RF phase is the standard deviation of 10 measurements. The $K_{CAL}$ can be evaluated in both cases from the data shown in Fig. 9, using Eq. 8, and for the conventional layout is equal to $7.37 \cdot 10^{11} \hbox {m/s}$, while for the non-conventional layout, $K_{CAL}$ is equal to $3.30 \cdot 10^{11} \hbox {m/s}$.

Figure 9 highlights a satisfying agreement between measurements, simulations, and theory (the maximum absolute error, between measurements and theory, is in the range between 0.019 and 0.34 mm). Furthermore, the bunch length evaluated using Eq. 19 is exactly the same, within experimental errors, for both configurations: $1.9 \pm 0.3 \, \hbox {ps}$. All the results are consistent with the theory prediction.

## Discussion

The non-conventional layout opens new opportunities with a strong impact on the accelerators physics community: (i) increase in the input dynamic range of the measurement, giving rise to a further enhancement in resolution and precision; and (ii) measurement of the correlations between the vertical plane and the longitudinal position, by varying the focusing power of the quadrupole.

For the latter point, the correlation terms can be picked up from Eq. 11, by assessing the difference between the two $\sigma _{y_{s,ON}}$ in phase opposition:

22 $$\tau = {\sigma ^2_{y_{s,ON}}(\phi )-\sigma ^2_{y_{s,ON}}(\phi +\pi )} =4M_{11}K_{CAL}(\phi )\sigma _{{y_{0}}{t_{0}}}+ 4M_{12}K_{CAL}(\phi )\sigma _{{y^{\prime}_{0}}t_{0}},$$

where $\tau$ is the sum of the correlation terms, each one multiplied by known factors. In the non-conventional layout, the two correlation terms have different dependence on the focal length of the quadrupole, making it possible to isolate and quantify their individual effects. In fact, the terms $M_{11}$ is multiplied by the correlation terms $\sigma _{y_{0}t_{0}}$, and the term $M_{12}$ is multiplied by the correlation terms $\sigma _{y^{\prime}_0t_0}$. This consideration is not valid in the case of the conventional layout^41^.

## Conclusion

In this paper, the effect on the bunch length measurement technique of additional focusing elements between the RF deflector and the screen has been analyzed and modeled. All the derived equations have been numerically validated by means of the ELEGANT code. Moreover, a preliminary experimental validation has been carried out at the CLEAR facility. A good agreement have been found for all the physical quantities, as well as in terms of accuracy and precision, between: theoretical predictions, simulations results, and measurements. From the theoretical derivation, the following conclusions are obtained: (i) a calibration factor with the same meaning as in the conventional layout can be defined in a sound way; (ii) the absence of additional terms which could introduce systematic errors is shown, (iii) the possibility of removing correlation effects is preserved, by first two independent measurements of the spot size with the RFD on in phase opposition, and then by assessing the average between their squared values, and (iv) thanks to the dependence of the beam sizes on the focal length a wider range of beam parameters can be exploited at the entrance of the RFD. Furthermore, a critical value of the focal length preventing the measurements to perform, and thus to be avoided, was identified. In conclusion, the non-conventional layout was validated satisfyingly.

Both the calibration factor and the $\sigma _{y_{s,OFF}}$ with RFD off depend on the focal length of the focusing element. For this reason, the optimum resolution is not necessarily obtained by minimizing the beam size on the screen with the RFD off, like in the conventional layout. While this implies a more complex experimental configuration, it opens up the possibility of a further enhancement in input dynamic range, making available further improvement of resolution and precision.

In the future, a full study, including a more comprehensive experimental validation campaign, will be carried out. First, the optimum focal length maximizing the resolution and the precision, is to be identified. Then, the achieved metrological performance is to be compared for the conventional and non-conventional layout. Besides, as discussed above in section “Discussion”, the additional advantage of measuring the correlations is to be further investigated and proved experimentally.
